# Bluish-Green Coloration of the Nipples: A Diagnostic Clue for Retroareolar Cysts

**DOI:** 10.3390/children12091224

**Published:** 2025-09-13

**Authors:** Tea Curic, Francesco Bellinato, Paolo Gisondi, Giampiero Girolomoni

**Affiliations:** Section of Dermatology and Venereology, Department of Medicine, University of Verona, 37129 Verona, Italypaolo.gisondi@univr.it (P.G.); giampiero.girolomoni@univr.it (G.G.)

**Keywords:** nipple, retroareolar cyst, bluish coloration

## Abstract

**Highlights:**

**What are the main findings?**

- Bilateral bluish-green retroareolar macules in a pubertal girl were diagnosed as benign retroareolar cysts through dermoscopic and ultrasound evaluation, with stability over one year of conservative management.

**What is the implication of the main finding?**

- Recognizing this benign presentation can prevent unnecessary diagnostic procedures and support conservative management, preserving normal pubertal breast development.

**Abstract:**

**Background/Objectives:** Bluish-green discoloration of the nipples in pubertal girls often poses a diagnostic dilemma. **Methods:** We describe the case of an 11-year-old girl who developed bilateral and symmetrical bluish-green macules in the retroareolar area over eight months. Dermoscopic and ultrasound examinations revealed benign retroareolar cysts, with no signs of malignancy or other alarming features. Differential diagnoses such as vascular malformations, hemangiomas, hematomas, and blue nevi were carefully considered and excluded based on clinical and imaging findings. **Results:** A diagnosis of bilateral retroareolar cysts was established. The patient underwent conservative management, and ultrasound follow-ups over one year showed no changes. **Conclusions:** This case underscores the importance of thorough evaluation to avoid unnecessary invasive procedures and to protect the delicate development of the pubertal breast.

## 1. Introduction

Bluish lesions appearing in the nipple area on pubertal girls present a significant diagnostic challenge. Accurate identification of this clinical picture is crucial for implementing appropriate conservative management, thereby preventing unwarranted diagnostic procedures like skin biopsies or extensive radiological examinations. These interventions could indeed negatively interfere with the normal anatomical and functional development of a particularly sensitive region. Although the prevalence of this condition is unknown, it is likely underestimated, as mild or self-limiting cases may go unreported or be misdiagnosed [1]. We report the case of an 11-year-old girl who was referred to our clinic with a bluish discoloration of the nipples caused by retroareolar cysts.

## 2. Case Presentation

**Figure 1:**
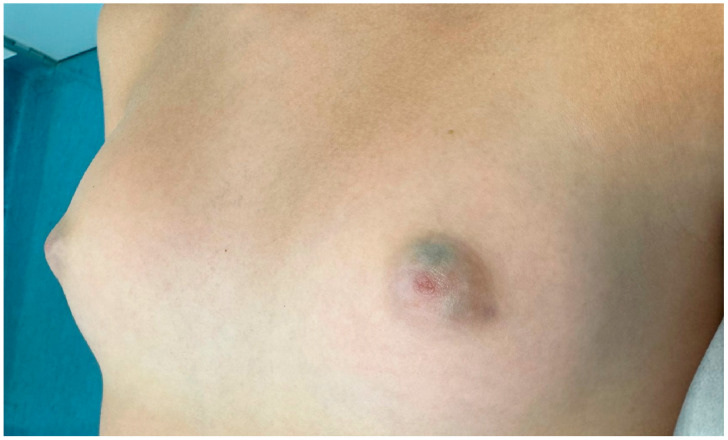
Bluish retroareolar cystic lesion.

**Figure 2:**
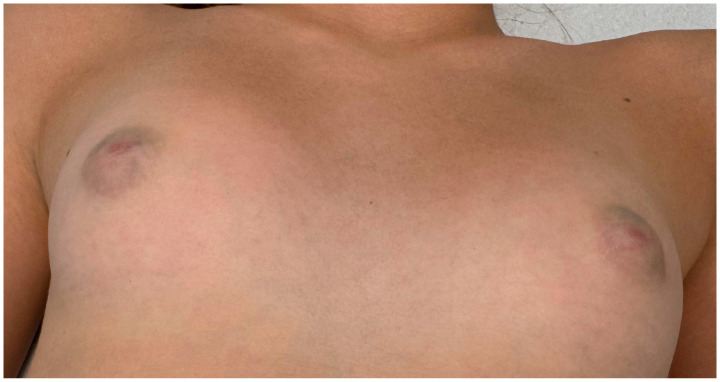
Bilaterality of lesions.

An 11-year-old girl presented to our clinic with bilateral and symmetrical bluish-green lesions observed in the retroareolar region. The peculiarity of the present case is the bilaterality of the lesions (Figure 1 and Figure 2).

**Figure 3:**
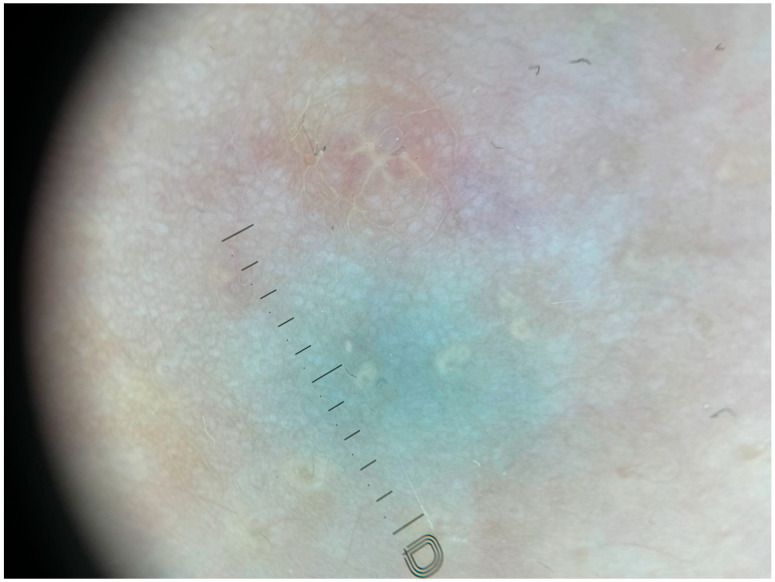
Dermoscopy showing unstructured greenish discoloration with blurred edges.

She reported that these lesions had first appeared approximately eight months prior to her visit. The patient was in good general health; she was not taking any medications and denied any prior surgical procedures. Upon physical examination, the lesions were noted to be superficial, soft, and smooth to palpation. The discoloration did not blanch during palpation. There was no evidence of discharge or solid thickening. Dermoscopic examination revealed a homogeneous, unstructured greenish discoloration with blurred edges (Figure 3).

**Figure 4:**
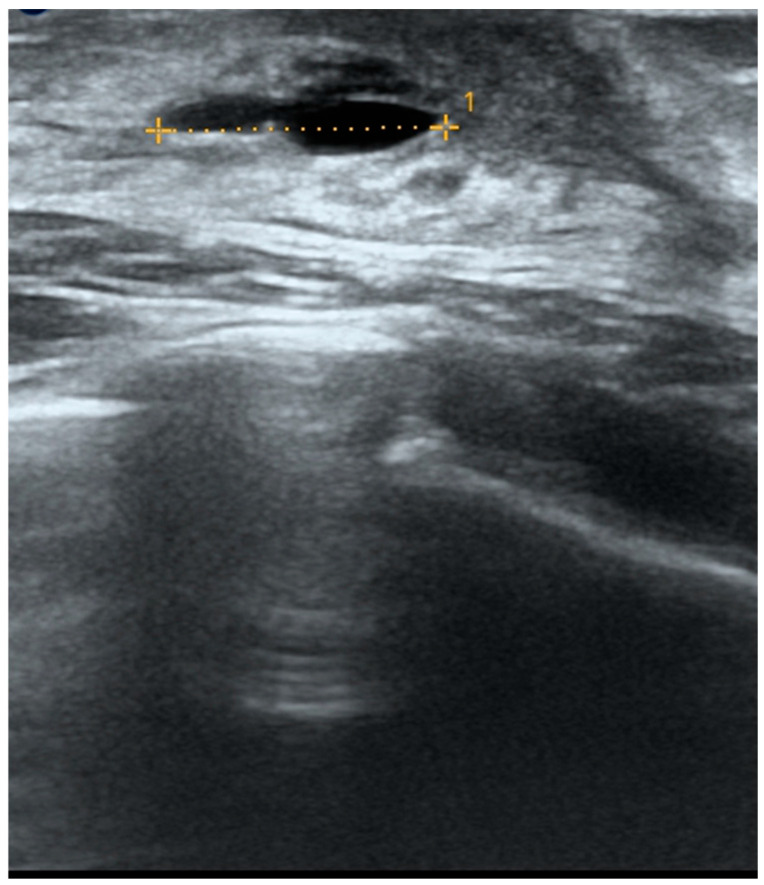
Baseline ultrasound (T0) showing an anechoic lesion with well-defined, regular margins (maximum diameter: 13 mm), consistent with a simple cyst.

An ultrasound (US) examination was therefore performed, selected for its non-invasive nature, absence of ionizing radiation, wide availability, and relatively low cost. In addition, US offers high sensitivity in the evaluation of superficial soft tissues and allows real-time imaging, making it particularly suitable for the assessment of developing breast tissue. The examination revealed developing mammary glands with a hypoechogenic retro- and peri-areolar echostructure and bilateral anechogenic formations, the largest of which was elongated and approximately 13 mm in diameter (Figure 4). Color Doppler evaluation was also performed and showed no internal vascular signals.

**Figure 5:**
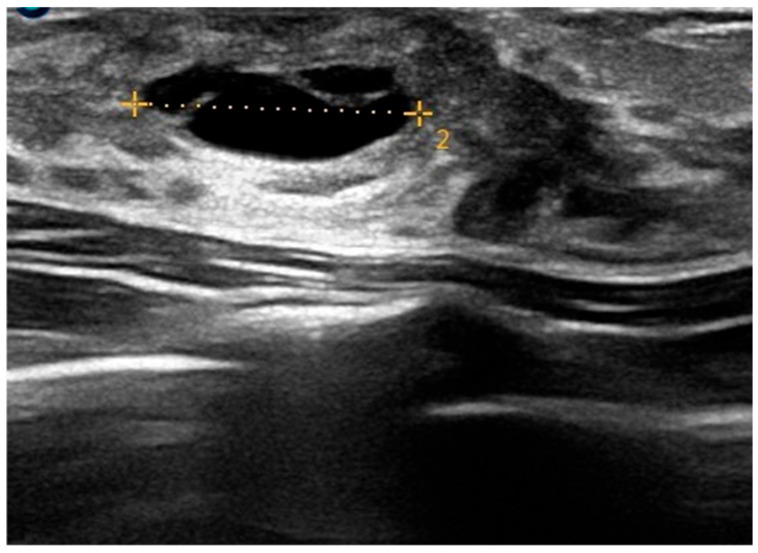
Ultrasound examination at 12-month follow-up confirming the presence of an anechoic lesion with regular margins, measuring approximately 15 mm in maximum diameter, stable over time.

Based on the findings, a diagnosis of bilateral retroareolar cysts was proposed, and conservative management was chosen. The cysts remained unchanged during subsequent follow-up visits. No symptoms such as mammary tenderness were reported. One year after the initial appearance of the lesions, a new ultrasound examination confirmed their substantial stability (Figure 5).

## 3. Discussion

Retroareolar cysts, also known as Montgomery cysts, occur due to obstruction of the Montgomery’s gland ducts [1,2]. The prevalence of this condition is unknown but likely underestimated. Few cases have been reported in the literature; a recent review article summarized nine cases (Table 1) [3].

Montgomery’s areolar tubercles are specialized sebaceous glands localized in the areolar region, structurally and functionally associated with the terminal portions of the lactiferous ducts that originate from the underlying mammary lobules. These glands contribute to areolar physiology, particularly during lactation, by secreting lubricating and antimicrobial substances. Montgomery’s areolar tubercles typically measure 2 mm or less in diameter [2].

The diagnosis of retroareolar cysts is based on clinical and dermoscopic evaluation, supported by a detailed medical history and confirmed by ultrasound examination (Table 2) [3].

The characteristic dermoscopic blue green discoloration of the cyst is likely related to the Tyndall effect. Management is generally conservative because the majority of cases resolve spontaneously with an excellent prognosis. The clinical manifestations largely depend on whether the cyst is infected or not. Infected cysts may present as a palpable mass accompanied by mastalgia and peri-areolar erythema. In contrast, noninfected (simple) cysts are often asymptomatic and may be incidentally detected [1].

Differential diagnosis may include vascular and lymphatic lesions, hematomas, and blue nevi [4]. Vascular lesions, such as deep haemangiomas, typically present early in life and exhibit distinctive dermoscopic features, including polymorphous vascular structures and dilated linear vessels [6]. Lymphatic malformations, by contrast, often appear as multiloculated, translucent vesicles and may fluctuate in size due to fluid accumulation or infection. Hematomas can mimic cystic or vascular lesions but are usually associated with a history of trauma and demonstrate characteristic imaging features on ultrasound. They typically resolve spontaneously within 2–3 weeks. The sonographic appearance of a hematoma varies depending on its age and the degree of internal liquefaction. In the acute phase, a hematoma typically appears as a hyperechoic focal collection with ill-defined margins. As the hematoma evolves, it undergoes progressive liquefaction, ultimately assuming the appearance of a complex cystic lesion with internal echogenic debris and fibrous septations [7]. Common blue nevi exhibit distinctive dermoscopic features that generally allow for confident and accurate diagnosis. These lesions typically present as homogeneously blue structures, ranging in color from blue-gray to steel-blue. The pigmentation is evenly distributed and sharply circumscribed, often appearing as a solitary, well-demarcated area without additional dermoscopic structures. Notably, common blue nevi lack features such as pigment networks, dots, globules, streaks, or vascular patterns [8]. Blue nevi, on ultrasound examination, typically do not appear as cystic lesions and do not present bilaterally; instead, they exhibit a characteristic hypoechoic, ‘dish-shaped’ pattern [9].

In the patient’s medical history, no prior surgical procedures were reported. However, had such procedures been present, alternative diagnostic hypotheses would have needed consideration. For instance, following a ductoscopy involving a tracer dye like methylene blue, it would be crucial to recognize the possibility of persistent blue discoloration on the nipple surface.

The same considerations apply to other dyes commonly used in surgical settings, such as patent blue and isosulfan blue, which are frequently employed in sentinel lymph node mapping. Gentian violet (crystal violet) is another dye with the potential to cause cutaneous staining and has been used in the treatment of nipple thrush. In some cases, cysts may present with unusual imaging features, including internal echoes, layered fluid levels, internal septa, or thickened walls, which can complicate diagnosis. In such situations, aspiration can help clarify the nature of the lesion and exclude other possibilities like a galactocele, an abscess, or a more complex cyst [7].

## 4. Conclusions

In summary, retroareolar cysts typically do not require intervention in the absence of inflammatory signs or secondary infection. In the presented case, the diagnosis was established through clinical dermoscopic examination and targeted high-frequency ultrasound, which confirmed the cystic nature of the lesion without features suggestive of malignancy. Given the typically benign and self-limiting nature of these lesions, preserving the integrity of the developing breast tissue is important. Invasive procedures, which could interfere with the natural development of the mammary glands, should be avoided whenever possible.

## Figures and Tables

**Table 1:** Clinical cases.

Patient	References	Age	Clinical Presentation
1	Sechi [4]	10 years 3 months	Two weeks’ history of monolateral blue cyst
2	Sechi [4]	10 years 6 months	Three-month history of bilateral blue lumps
3	Rusiñol [5]	11 years	Bluish cyst, asymptomatic, 1 year duration
4	Rusiñol [5]	12 years	Asymptomatic bluish cyst, 3 years duration
5	Huneeus [2]	Not reported	Bluish appearance of retroareolar cyst
6	Huneeus [2]	Not reported	Bluish appearance of retroareolar cyst
7	Mioso [3]	10 years 2 months	Monolateral green-blue cyst, 2 weeks duration
8	Mioso [3]	11 years 4 months	Bilateral blue lumps, 1 year duration
9	Mioso [3]	11 years 7 months	Monolateral blue cyst, 1-year duration
10	Our case	11 years	Eight-month history of bilateral symmetrical bluish-green cyst

**Table 2:** Differential diagnosis.

Differential Diagnosis	Clinical Features	Dermoscopic Features	Ultrasound Features	Age/Notes
Retroareolar cysts	Superficial, soft, smooth and bluish lesions	Homogeneous, unstructured greenish discoloration with blurred edges	Anechoic, oval, thin-walled mass with posterior enhancement; no Doppler flow	Prepuberal age
Deep haemangiomas	Bluish-purplish swelling or mass	Normal or bluish skin, sometimes with a telangiectatic hue	Well-defined, hypoechoic, hypervascular structure with rapid flow (growth phase); more echogenic with reduced vessel size/density (involution phase)	Early in life. Consider presence of superficial hemangioma component.
Lymphatic malformations	Multiloculated, translucent vesicles	Yellowish-white lacunae separated by a thin, pale, whitish area	Well-defined, hyperechoic lesion with heterogeneous texture; hypervascular on color Doppler	Early in life
Hematomas	Painful lumps or bumps characterized by skin discoloration	Violaceous discoloration with blurred edges; evolves into green/yellowish hues over time	Hyperechoic with ill-defined margins acutely; progresses to complex cystic lesion with internal debris and septations	Resolve spontaneously within 2–3 weeks
Common blue nevi	Sharply circumscribed, homogeneously blue lesions	Free from pigment networks, dots, globules, streaks, or vascular patterns	Hypoechoic, dish-shaped pattern	Non-bilateral
Surgical dye-related discoloration	Persistent blue discoloration on the nipple surface	Homogeneous bluish discoloration with blurred margins	Negative	Consider surgical history

## Data Availability

The data presented in this study are available on request from the corresponding author due to privacy, legal and ethical reasons.
